# Split‐Cas9‐based targeted gene editing and nanobody‐mediated proteolysis‐targeting chimeras optogenetically coordinated regulation of Survivin to control the fate of cancer cells

**DOI:** 10.1002/ctm2.1382

**Published:** 2023-08-24

**Authors:** Changping Deng, Shihui Li, Yuping Liu, Wen Bao, Chengnan Xu, Wenyun Zheng, Meiyan Wang, Xingyuan Ma

**Affiliations:** ^1^ State Key Laboratory of Bioreactor Engineering East China University of Science and Technology Shanghai P. R. China; ^2^ Shanghai Key Laboratory of New Drug Design School of Pharmacy East China University of Science and Technology Shanghai P. R. China; ^3^ Synthetic Biology and Biomedical Engineering Laboratory Biomedical Synthetic Biology Research Center, Shanghai Key Laboratory of Regulatory Biology Institute of Biomedical Sciences and School of Life Sciences East China Normal University Shanghai P. R. China

**Keywords:** Coordinated regulating to Survivin, Fate of cancer cells, Nanobody targeted degradation, Photoactivatable proteolysis and editing

## Abstract

**Background:**

Precise regulation of partial critical proteins in cancer cells, such as anti‐apoptotic proteins, is one of the crucial strategies for treating cancer and discovering related molecular mechanisms. Still, it is also challenging in actual research and practice. The widely used CRISPR/Cas9‐based gene editing technology and proteolysis‐targeting chimeras (PROTACs) have played an essential role in regulating gene expression and protein function in cells. However, the accuracy and controllability of their targeting remain necessary.

**Methods:**

Construction of UMUC‐3‐EGFP stable transgenic cell lines using the Sleeping Beauty system, Flow cytometry, quantitative real‐time PCR, western blot, fluorescence microplate reader and fluorescence inverted microscope analysis of EGFP intensity. Characterization of Survivin inhibition was done by using Annexin V‐FITC/PI apoptosis, calcein/PI/DAPI cell viability/cytotoxicity assay, cloning formation assay and scratch assay. The cell‐derived xenograft (CDX) model was constructed to assess the in vivo effects of reducing Survivin expression.

**Results:**

Herein, we established a synergistic control platform that coordinated photoactivatable split‐Cas9 targeted gene editing and light‐induced protein degradation, on which the *Survivin* gene in the nucleus was controllably edited by blue light irradiation (named paCas9‐Survivin) and simultaneously the Survivin protein in the cytoplasm was degraded precisely by a nanobody‐mediated target (named paProtacL‐Survivin). Meanwhile, in vitro experiments demonstrated that reducing Survivin expression could effectively promote apoptosis and decrease the proliferation and migration of bladder cancerous cells. Furthermore, the CDX model was constructed using UMUC‐3 cell lines, results from animal studies indicated that both the paCas9‐Survivin system and paProtacL‐Survivin significantly inhibited tumour growth, with higher inhibition rates when combined.

**Conclusions:**

In short, the coordinated regulatory strategies and combinable technology platforms offer clear advantages in controllability and targeting, as well as an excellent reference value and universal applicability in controlling the fate of cancer cells through multi‐level regulation of key intracellular factors.

## Graphical abstract

- The photoactivatable PROTACs‐Like (paProtacL) target protein via nanobodies and can controllably induce degradation of POI (protein of interest) under blue light.
- The photoactivatable Cas9 (paCas9) system can edit GOI (gene of interest) in the nucleus in a controlled manner under blue light irradiation.
- When paProtacL is combined with the paCas9 system, it can minimize the expression of the target protein.

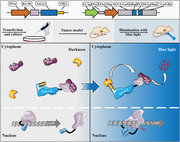

## 1 INTRODUCTION

Several key protein molecules play an essential role in the apoptosis, proliferation and migration of cancerous cells and these proteins are also in abnormal expression compared with normal tissues.^1^, ^2^, ^3^, ^4^ To better verify and study the function of these protein molecules, interference with protein expression is an effective strategy.^5^ The emergence and development of CRISPR/Cas9 technology provide an effective method for studying the function of specific genes.^6^ Although CRISPR/Cas9 can edit the target gene, it still has no direct edit on the protein product of the gene,^7^ and there are side‐effects such as off‐target edits and genotoxicity.^8^ Also, CRISPR‐Cas9 can generate large deletions and complex rearrangements, as well as adeno‐associated virus (AAV) or LINE‐1 retrotransposon insertions.^9^, ^10^, ^11^, ^12^ At this stage, proteolysis‐targeting chimeras (PROTACs) are a skill that employs the ubiquitin‐proteasome system to disintegrate the target protein.^13^ It consists of three specific elements, E3 ubiquitin ligase ligand, target protein ligand and linker.^14^ By recruiting the targeted protein to the vicinity of E3 ubiquitin ligase, the multi‐ubiquitination of the targeted protein is realized and finally degraded by the proteasome.^15^, ^16^, ^17^ Although the target protein is at the degradation level, its gene is still active at the transcription level.^16^, ^18^ Through comparison, although it can be found that CRISPR/Cas9 and PROTACs all have several shortcomings,^8^, ^16^, ^18^ targeting proteins using both systems synergistically can result in a rapid reduction in target protein expression. Certainly, effective degradation of abnormally expressed proteins is also a feasible strategy for cancer treatment.^19^

Precise control of the expression of key genes in cancer is essential for tumour treatment and cell fate.^20^ Therefore, it is urgent to construct a controllable platform for regulating gene expression. Light can be used as an ideal inducer due to its low toxicity, easy access, easy manipulation, high spatiotemporal resolution and other advantages.^21^, ^22^, ^23^ Nihongaki et al.^21^ developed the photoactivatable Cas9 (paCas9) system for gene editing, which divided the complete Cas9 protein into two parts in space, respectively fused with nMag and pMag proteins, which could form heterodimers under blue light irradiation, thus, combining split‐Cas9 into one, and then efficiently and controllably played the editing function. As designed, the split‐Cas9 protein dissociated in the nucleus under dark conditions, and when illuminated with blue light, nMag dimerized with pMag to enable the intact Cas9 protein to perform its shearing function. Meanwhile, according to the properties of AsLov2 (from *Avena sativa*), the target protein can be fused at the C‐terminus of the Jα helix, and the release of the target protein occluded in the LOV domain can be regulated by blue light.^24^, ^25^ Hence, AsLov2 can well exist as a controllable ‘linker’. The ligand of von Hippel‐Lindau (VHLL) contains only seven amino acids ALAPYIP,^26^, ^27^ but can effectively enrich E3 ligase, and the short ligand favours concealment by the C‐terminal Jα helix of AsLov2.^28^ As the ‘warhead’ for PROTACs, nanobody can bind with target protein.^29^, ^30^ The fusion of these three components constructed a photoactivatable PROTACs‐Like (paProtacL) degradation system. It works as designed, where the E3 ligand VHLL was hidden by a tightly folded Jα helix under dark conditions. Under blue light irradiation, the Jα helix loosened, releasing VHLL to bind to the E3 ligase and degrade the protein of interest (POI) via the ubiquitination‐dependent degradation pathway.

Survivin (also called BIRC5), a strategic and fundamental protein molecule in cancer, mainly plays the role of anti‐apoptosis and is exceedingly apparent in its expression in all tumour cells including bladder cancer.^31^, ^32^, ^33^ Therefore, Survivin has been projected as a striking target for innovative anti‐cancer interventions. Lactose‐derived branched cationic biopolymer delivery system emerged from the work of Qi et al.^34^ in transmitting Cas9 for editing the *Survivin* gene to cure hepatocellular carcinoma. Hu et al.^33^ established a degradation platform that could constrain Survivin at both transcriptional and protein levels based on ferritin nanocage. Meanwhile, Deng et al.^32^ used the Tet‐off system to regulate Survivin expression precisely and found that Survivin might mediate the development of chemotherapy resistance. All the above studies showed that inhibiting the expression of Survivin could be used to treat cancers and reduce the generation of chemoresistance. Meanwhile, the choice of an efficient delivery system is necessary for better gene delivery.^35^, ^36^ Lentiviruses have a wide range of hosts, a large gene capacity, high infection efficiency and long‐term stable expression by integrating genes into host cells.^37^ Recently, it has been widely used in studying genetic mechanisms and disease development.^35^, ^36^, ^37^ Hence, lentiviral vectors can be chosen to deliver the paCas9 and paProtacL systems to reduce Survivin expression in cancers effectively.

Herein, we established a platform that could minimize specific gene expression by combining the paCas9‐GOI (gene of interest) and paProtacL‐POI systems. Survivin protein, as an example, the effective sgRNA targeting *Survivin* and a nanobody Nb4A capable of specifically binding Survivin protein were selected.^32^, ^38^ Under the irradiation of blue light, the expression of Survivin was controllably inhibited. The platform not only makes it easier to study the proteins with unknown functions, but also provides an effective strategy for degrading protein molecules that are aberrantly expressed in cancer, in addition to controlling the fate of cancer cells.

## 2 MATERIALS AND METHODS

### 2.1 Cell lines and cell culture

The cells derived from cancerous human bladder include UMUC‐3, 5637, T24, SW780 and RT4 cells and were bought from the American Type Culture Collection (ATCC). UMUC‐3/SW780/RT4 cell lines were developed in DMEM, and 5637/T24 were grown up in 1640 medium, which all augmented with 10% fetal bovine serum (Invitrogen) and 1% antibiotics (Penicillin/Streptomycin/Amphotericin B) (Solarbio) in a 5% CO_2_ incubator at 37°C. The HEK‐293T (embryonic kidney) cell line was also bought from ATCC and cultured in DMEM following the manufacturer's protocol.

### 2.2 Construction of stable transfer cell lines

The initial cells were UMUC‐3 bladder cancer cells. The plasmid system used to construct the stable transfer cell lines was Sleeping Beauty. The EGFP‐containing plasmid system was constructed and co‐transfected with a transposase plasmid into UMUC‐3 cells, and a stable EGFP‐transfected cell line, UMUC‐3‐EGFP, was obtained through multiple rounds of puromycin screening. Later, genomic polymerase chain reaction (PCR) identification and flow cytometry were performed to determine whether it was a stably transfected cell line.

### 2.3 Bioinformatics analysis

Analysis of *Survivin* gene expression levels in pan‐cancer (abbreviations and full names in Supporting Information Table S1) by the cancer genome atlas (TCGA) online database (http://ualcan.path.uab.edu/analysis.html). Immediately after, the *Survivin* gene expression was analysed in bladder urothelial carcinoma (BLCA) and showed the *Survivin* gene expression in BLCA based on distinct stages of cancer. Meanwhile, Survivin protein expression in normal and cancerous bladder tissues was scrutinized by the human protein atlas (https://www.proteinatlas.org).

### 2.4 Western‐blot analysis

Washing of the treated cells was carried out in phosphate buffered saline (PBS) and lysed in RIPA buffer (high) (Solarbio, Beijing, China), which contained 1 mM phenylmethylsulfonyl fluoride, Solarbio. Calculation regarding protein concentration was done utilizing the bicinchoninic acid protein assay kit (Sangon Biotech). Equivalent amounts of whole protein extract (30 µg) were electrophoresed on SDS‐polyacrylamide gels and then transferred to polyvinylidene difluoride membranes (pore size: 0.22 µm) using the Yeasen Blot‐transfer unit. After blocking with 5% milk, incubated the membranes overnight with specific primary antibodies against Survivin (Mouse/IgG1; 1:3,000; Proteintech), glyceraldehyde‐3‐phosphate dehydrogenase (Mouse/IgG2b; 1:50,000; Proteintech), His (Mouse/IgG1; 1:10,000; Proteintech), Flag (Rabbit/IgG; 1:10,000; Bioworld), HA (Human influenza hemagglutinin) (Rabbit/IgG; 1:3,000; Bioworld), EGFP (Mouse/IgG1; 1:2000; Leading Biology) at 4°C. The membranes were later incubated with horseradish peroxidase‐conjugated secondary antibody (Goat Anti‐Rabbit IgG (H+L) or Goat Anti‐Mouse IgG (H+L); 1:6000; Proteintech) for 2 h on a horizontal oscillator at room temperature. Products were generated on film by the chemiluminescence kit purchased from Sangon Biotech and quantitated by ImageJ software.^32^, ^33^, ^39^

### 2.5 Plasmid construction

The initial carrier was pLVX‐EF1α‐IRES‐Puro, and the working plasmid was pLVX‐HA‐Nb4A‐AsLov2‐VHLL‐IRES‐Puro (the expression cassette was located between *Bam*H I and *Eco*r I), named paProtacL‐Survivin. Nb4A was a specific nanobody against Survivin that was previously reported and studied in our laboratory,^38^ and VHLL was the ligand of von Hippel‐Lindau (VHL). pLVX‐HA‐LaG16‐AsLov2‐VHLL‐IRES‐Puro (enzyme cut site as above), LaG16 was the previously reported nanobody to EGFP.^40^ pLVX‐U6‐sgRNA‐Survivin‐EF1α‐Flag‐NLS‐N‐Cas9‐nMag‐P2A‐NLS‐pMag‐C‐Cas9‐His (the U6‐sgRNA‐Survivin expression cassette was located between *Cla* I and *Nde* I, Flag‐NLS‐N‐Cas9‐nMag‐P2A‐NLS‐pMag‐C‐Cas9‐His expression cassette was located between *Bam*H I and *Sma* I), named paCas9‐Survivin. The N‐Cas9 was the residue 2−713 of the full‐length Cas9, and the C‐Cas9 was the residues 714–1368 of the full‐length Cas9. pMag and nMag, as photoinducible dimerization domains, could heterodimerize upon blue light irradiation. All fragment‐to‐fragment ligation by homologous recombinase was purchased from Transgen Biotech. The sgRNA primers utilized in the current study were listed in Supporting Information Table S2, and the amino acid sequences of all genes were shown in Supporting Information Table S3.

### 2.6 Packaging of recombinant lentiviruses

The constructed working plasmid, psPAX2 and pMD2.0G, were extracted by the application of an endotoxin‐free plasmid extracting kit (TIANGEN), then co‐transfection of HEK‐293T cells with the three plasmids by Lipofectamine 3000 (Thermo Fisher) coinciding with instructions of manufacturer. After 8 h of transfection, the complete medium superseded the former medium. After 48 and 72 h of culture, the supernatant possessing lentivirus particles was collected and centrifuged at 250 *g* for 5 min to remove the cell debris. Cells and debris were additionally dislodged by filtering the supernatant (30‐kDa MWCO, Millipore, Merck), and its volume was condensed to achieve an escalated lentivirus concentration. According to the literature,^41^ the virus titre in HEK‐293T cells was measured by means of the end‐point dilution assay. And then, the multiplicity of infection (MOI) = 20 or 10 was selected for lentiviral transfection.

### 2.7 Quantitative real‐time PCR (qRT‐PCR)

The total RNA extraction and reverse transcription kits from Tiangen Biotech Co., Ltd. were used to obtain cDNA templates for qRT‐PCR. Meanwhile, the qRT‐PCR assay was performed on the LightCycler 96 real‐time PCR instrument (BioRad) employing a SuperReal PreMix Plus (SYBR Green) kit. Based on previous reports,^32^ the β‐actin was selected as an internal control, and all primer pairs were included in Supporting Information Table S2. The comparative Ct (^ΔΔ^ *Ct*) method was utilized to quantify the gene expression.

### 2.8 Cell apoptosis assay

Referring to the previously reported method,^42^ UMUC‐3 and 5637 cells were inoculated into 6‐well plates at 5.0 × 10^5^ cells per well, respectively, and incubated with packaged lentiviruses paCas9‐Survivin and paProtacL‐Survivin separately or in combination for 48 h, and then followed by blue light irradiation (1.2 W/m^2^). After 48 h of incubation under blue light irradiation, the apoptosis rate in the above‐treated cells was measured by the flow cytometry (Beckman Coulter) via an Annexin V‐FITC/PI Apoptosis Kit (Vazyme) in relevance to the manufacturer's protocol. Each experiment was repeated three times.

### 2.9 Cloning formation assay

UMUC‐3 and 5637 cells were respectively implanted into a 6‐well plate at 2.0 × 10^3^ cells per well and treated with packaged lentiviruses paCas9‐Survivin and paProtacL‐Survivin separately or in combination for 48 h and then exposed to 1.2 W/m^2^ blue light for another 48 h. The medium was altered every 2 days subsequently. After 14 days, washing of the cells was ensured two times with PBS and fixed with a formaldehyde solution (4%). All treated cells were stained and imaged using crystal violet solution (0.1%). The repetition experiment was brought about three times.

### 2.10 Cell migration assay

The wound‐healing assay has been exploited for the analysis of cell migration. Concisely, cells were added in 12‐well plates at equal density and developed to 90% confluency. Also, treatment with packaged lentiviruses paCas9‐Survivin and paProtacL‐Survivin separately or in combination for 48 h, the medium containing 1% fetal bovine serum was then replaced and the cells were scratched. Subsequently, the treated cells were uncovered by blue light irradiation (1.2 W/m^2^) for treatment. The sterile pipette tip was used for generating artificial gaps. Wound areas were highlighted and photographed with a digital camera system. The distance of cell migration was determined by the software program HMIAS‐2000. Individual experiments were repeated three times.

### 2.11 Cell viability assays

UMUC‐3 and 5637 cells were respectively plated onto 12 wells at a density of 5.0 × 10^4^ cells per well at 37°C in a 5% CO_2_ incubator. After 24 h, treated with packaged lentiviruses paCas9‐Survivin and paProtacL‐Survivin separately or in combination for 48 h, and then illuminated by 1.2 W/m^2^ blue light for another 48 h. Immediately after, the medium was aspirated and rinsed gently twice with pre‐chilled PBS. Subsequently, the living and dead cells were identified by calcein/PI cell viability/cytotoxicity Assay Kit (Beyotime) and DAPI staining reagent (Solarbio), consistent with the manufacturer's guidelines. Then, the cells were examined by a fluorescent microscope (Olympus). FITC channel (λex 488 nm and λem 525 nm), PI channel (λex 535 nm and λem 615 nm) and DAPI channel (λex 364 nm and λem 454 nm).

### 2.12 In vivo anticancer effect evaluation

Female BALB/c nude mice 5‐week‐old were unsystematically allocated into the control and experimental groups (n = 5 for each group) (Nanjing Junke Biological Co., Ltd.). There were three experimental groups and one control group including paCas9‐Survivin alone, paProtacL‐Survivin alone and paCas9‐Survivin combined with paProtacL‐Survivin. The control group was inoculated with primary UMUC‐3 cells, while the experimental group was inoculated with cells treated with corresponding lentivirus‐infected UMUC‐3 cells. The cells were already included in seeding the experimental mice with the paCas9‐Survivin and/or paProtacL‐Survivin system. For the cell‐derived xenograft (CDX) model, 1.0 × 10^7^ cells in 100 µL DMEM (non‐resistant, serum‐free and phenol red‐free) were injected at the right side of the back of each mouse, by following the formula volume (mm^3^) = (length) × (width)^2^/2, the tumour size was determined after the interval of 3 days. All experimental groups of mice were well‐lighted from above through a blue LED lamp (90 mW/cm^2^) for 8 h every 3 days. Also, all mice's body weights were measured 3 days apart. Mice were euthanized 15 days after light exposure, and tumour sections were embedded, hematoxylin and eosin (HE), and immunohistochemistry (IHC) stained (Shanghai RIBIOLOGY Technology Co., Ltd.).

### 2.13 Statistical analyses

Data were expressed as means ± SD. Significance tests were performed by conducting one‐way ANOVA using the GraphPad Prism 8.0 software (GraphPad Software, La Jolla, CA). *p*‐value < 0.05 was thought‐out statistically significant (*p < 0.05, **p < 0.01, ***p < 0.001, ns means not significant).

## 3 RESULTS

### 3.1 Design, construction and test of controlled protein expression systems

**FIGURE 1:**
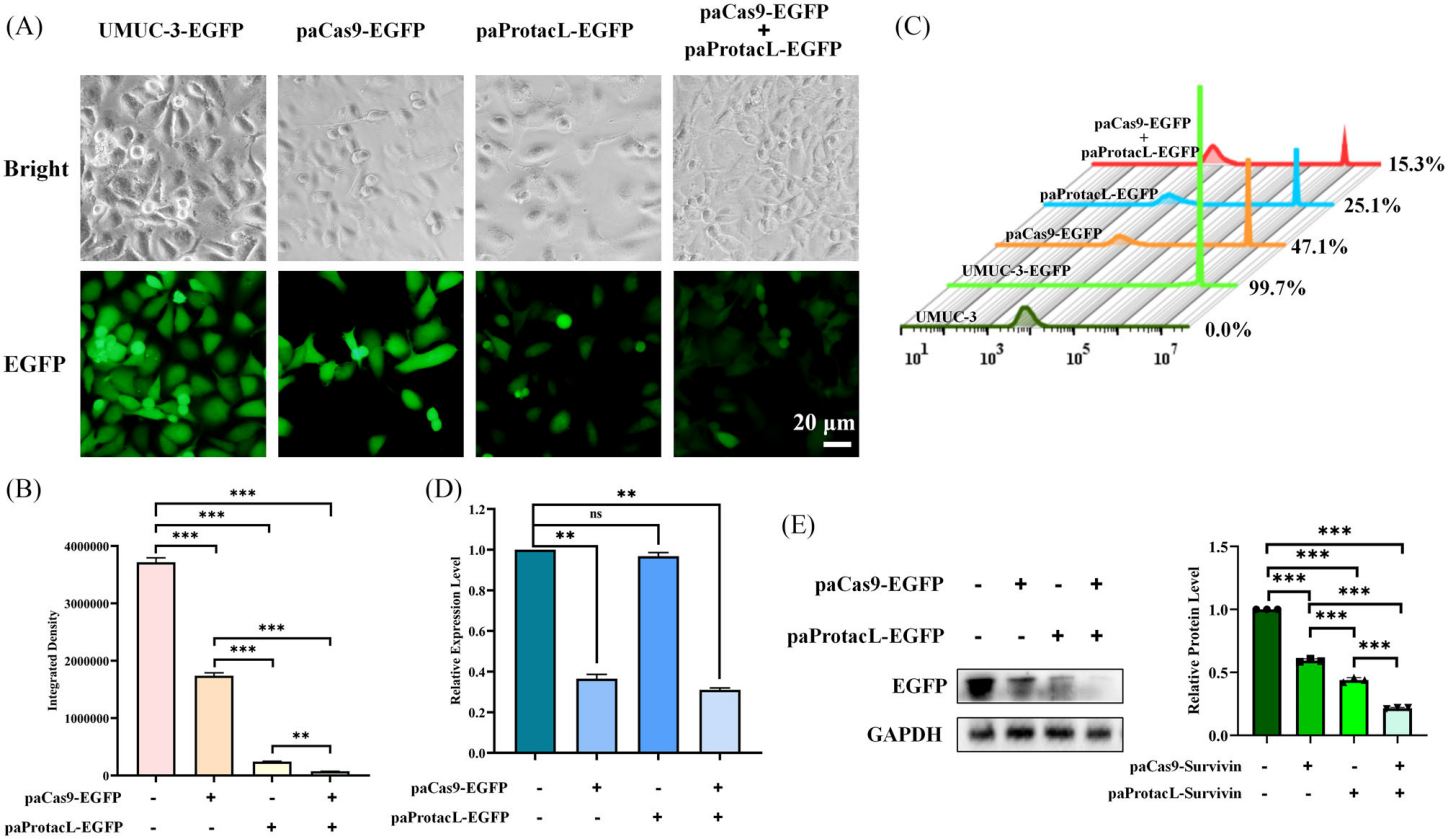
Combined degradation of EGFP protein by paProtacL‐EGFP and paCas9‐EGFP after 48 h of blue‐light irradiation. (A) Degradation of EGFP protein in UMUC‐3‐EGFP cells by paProtacL‐EGFP and paCas9‐EGFP alone or in combination were observed by fluorescence microscopy. (B) Fluorescence intensity of UMUC‐3‐EGFP after treatment with paProtacL‐EGFP and paCas9‐EGFP alone or in combination were analysed by ImageJ software. (C) Fluorescence levels of UMUC‐3‐EGFP after treatment with paProtacL‐EGFP and paCas9‐EGFP alone or in combination were measured by flow cytometry. (D) The mRNA levels of *E* *G* *F* *P* were detected by qRT‐PCR in the differently treated UMUC‐3‐EGFP cell lines. (E) The EGFP protein expression level in UMUC‐3‐EGFP cells after treatment with paProtacL‐EGFP and paCas9‐EGFP alone or in combination were detected by western blot. MOI = 20. Scale bar: 20 µm.

On the one hand, to perform controlled editing of specific genes, we chose the light‐controlled split‐Cas9 approach for targeting gene editing. According to the method reported by Nihongaki,^21^ nMag and pMag were selected as blue‐light receptor proteins, which could form heterodimers in response to blue light. Meanwhile, we picked the most efficient split‐Cas9 pair. That is, amino acids 2−713 of Cas9 are known as N‐Cas9 and 714–1368 as C‐Cas9. Also, sgRNAs were selected according to previous reports.^32^, ^43^ This method was named paCas9‐GOI. On the other hand, PROTACs were used to degrade hard‐to‐drug protein targets. We imitated the format of PROTACs by choosing nanobodies as ‘warheads’ to target the selected protein and VHLL to connect to E3 ligases. AsLov2 acted as a blue‐light receptor and used its C‐terminal property to connect E3 ligands. Identically, this was called paProtacL‐POI. As a result, combining these two techniques for the same gene could lead to more robust suppression. Taking the EGFP protein as an example, we constructed a UMUC‐3‐EGFP stable transfer cell line of bladder cancer that stably expresses EGFP (Supporting Information Figure S1). The pLVX‐HA‐LaG16‐AsLov2‐VHLL‐IRES‐Puro and pLVX‐U6‐sgRNA‐EGFP‐EF1α‐Flag‐NLS‐N‐Cas9‐nMag‐P2A‐NLS‐pMag‐C‐Cas9‐His lentiviral particles were added individually or in combination in 12‐well plate for 48 h. Immediately after the blue light exposure for 48 h, it could be tested. The combination of the two could be found by fluorescence microscopy to minimize green fluorescence (Figure 1A), and the ability of paProtacL‐EGFP to inhibit green fluorescence was more substantial than that of paCas9‐EGFP (Figure 1B). After 48 h of blue light irradiation, the fluorescence rate was 15.3% for the combination, 25.1% for paProtacL‐EGFP and 47.1% for paCas9‐EGFP by flow cytometry analysis (Figure 1C). In addition, the outcomes of qRT‐PCR exhibited that the paCas9‐EGFP system could remarkably interfere with the expression of *E* *G* *F* *P* at the transcriptional level, while paProtacL‐EGFP had essentially no effect on the mRNA expression of *E* *G* *F* *P* (Figure 1D). Meanwhile, the western blot assay of EGFP protein expression in the treated cells showed that the EGFP protein expression level decreased to 21.6% of the control when the two were combined (Figure 1E). The results above indicated that we constructed a controlled and highly inhibited degradation platform.

### 3.2 Survivin was selected as the target protein for degradation by bioinformatics analysis

**FIGURE 2:**
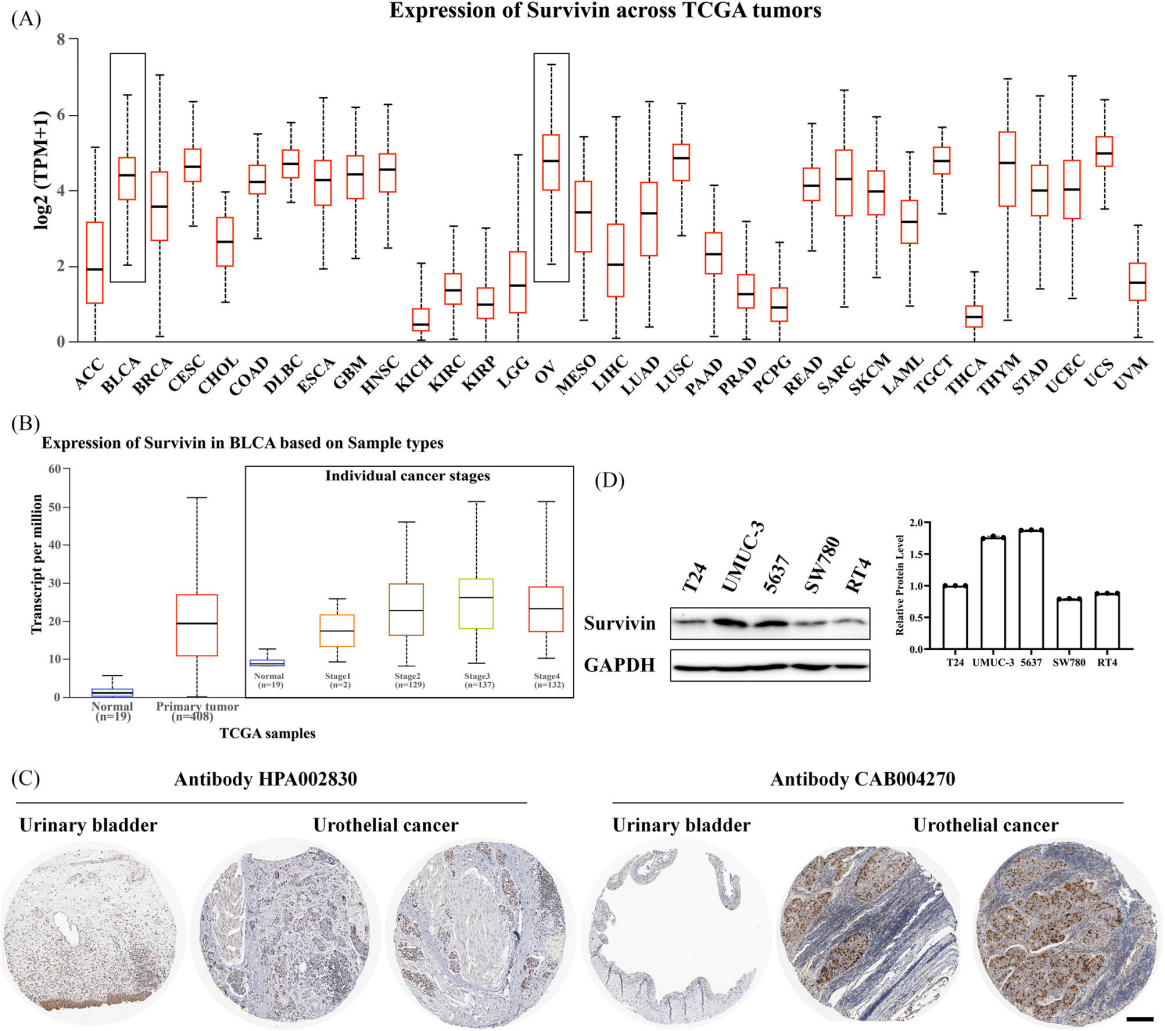
Bioinformatics analysis of Survivin expression in cancers. (A) Analysis of *Survivin* gene expression levels in pan‐cancer by TCGA database. (B) Analysis of *Survivin* gene expression levels in bladder urothelial carcinoma based on sample types and individual cancer stages. (C) Analysis of Survivin protein expression levels under different antibody staining by human protein atlas database. (D) Validation of Survivin protein expression levels in various bladder cancer cells by western blot. Scale bar: 200 µm.

*Survivin* gene expression level in pan‐cancer was investigated according to the online TCGA database. The results showed that *Survivin* was highly expressed in BLCA and highest in ovarian serous cystadenocarcinoma (OV) (Figure 2A). In BLCA, *Survivin* was conceived to be highly expressed in cancerous tissues compared to normal tissues, and the expression level remained higher than in normal tissues in different stages of BLCA (Figure 2B). Meanwhile, analysis of the Survivin protein expression level in human protein atlas presented that it was extraordinary in bladder cancer tissues and comparatively low in normal tissues, as demonstrated by different antibody staining (Figure 2C). In addition, several bladder cancer cells (T24, UMUC‐3, 5637, SW780 and RT4) available in the laboratory were selected to detect the expression level of the Survivin protein, and the western blot result demonstrated that higher levels of Survivin protein expression in UMUC‐3 and 5637 cells (Figure 2D). Therefore, the two cell lines were selected for further experiments.

### 3.3 Inhibition of Survivin protein by paProtacL‐Survivin and paCas9‐Survivin, respectively

**FIGURE 3:**
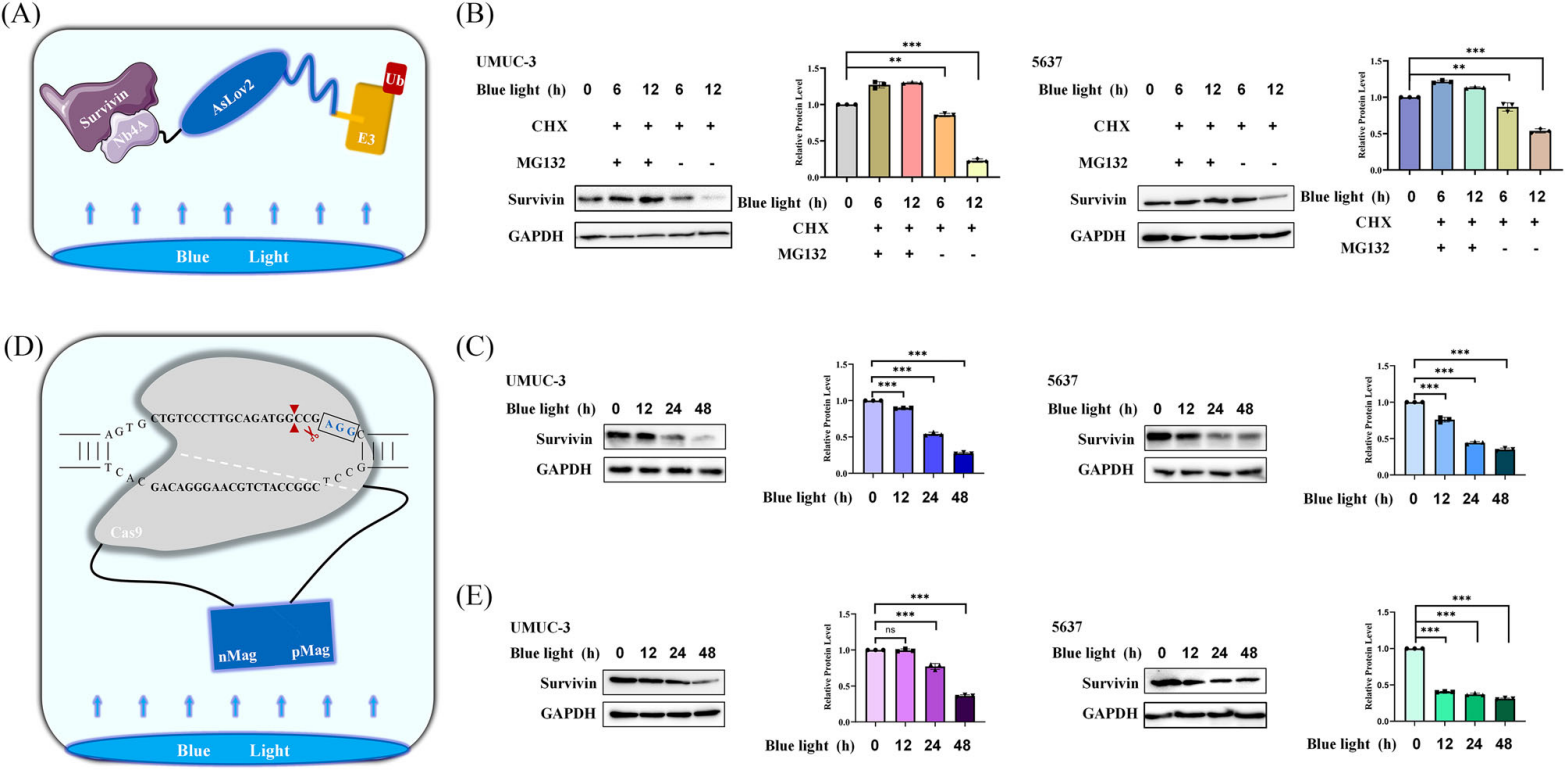
Inhibition levels of Survivin protein by paProtacL‐Survivin and paCas9‐Survivin, respectively. (A) Pattern diagram of paProtacL‐Survivin degradation of Survivin. (B) Detection of Survivin protein expression levels after treatment of UMUC‐3 and 5637 cells after transfection with pProtacL‐Survivin by blue‐light irradiation, CHX and MG132. (C) Survivin protein expression levels were detected in UMUC‐3 and 5637 cells after transfection with pProtacL‐Survivin by blue‐light irradiation for 48 h. (D) Diagram of how paCas9‐Survivin works. (E) Survivin protein expression levels were detected in UMUC‐3 and 5637 cells after transfection with paCas9‐Survivin by blue light irradiation for 48 h. CHX: Cycloheximide, mainly used as a protein biochemical synthesis inhibitor. MG132: Inhibits protein degradation mainly by inhibiting proteasome‐dependent degradation pathway. MOI = 20.

The working mode diagram of paProtacL‐Survivin is shown in Figure 3A. Nb4A was a nanobody to Survivin previously screened in the laboratory and could bind effectively to Survivin protein.^38^ Under blue‐light irradiation, the C‐terminus of AsLov2 exposed VHLL, which was able to attract E3 ligase in the cytoplasm, which in turn degraded Survivin protein. To verify that it was through the ubiquitination degradation pathway, the expression level of Survivin protein was detected after treatment of UMUC‐3 and 5637 cells with cycloheximide (CHX) (20 µg/mL), MG132 (5 µg/mL) and blue light. The western‐blot analysis implied that the expression level of Survivin protein was not remarkably different from the control when the three were present together, while only CHX and blue light were present, the Survivin protein expression level decreased in UMUC‐3 and 5637 cells with increasing treatment time (Figure 3B). The results above suggested that the degradation of Survivin was via the ubiquitinated degradation pathway. Moreover, when only blue‐light treatment was available, the expression level of Survivin protein in UMUC‐3 and 5637 cells could be found to decrease with time (Figure 3C). Meanwhile, in the paCas9‐Survivin system (Figure 3D), the selection of sgRNA was based on the previously reported,^32^ which was 5′‐CTGTCCCTTGCAGATGGCCG‐3′. Under blue‐light irradiation, the interacting nMag/pMag made the split‐Cas9 proteins merge into one and then edited the *Survivin* gene under the guidance of sgRNA. With the prolonged exposure time to blue light, the expression level of Survivin protein progressively decreased (Figure 3E).

### 3.4 paProtacL‐Survivin combined with paCas9‐Survivin to inhibit Survivin protein

**FIGURE 4:**
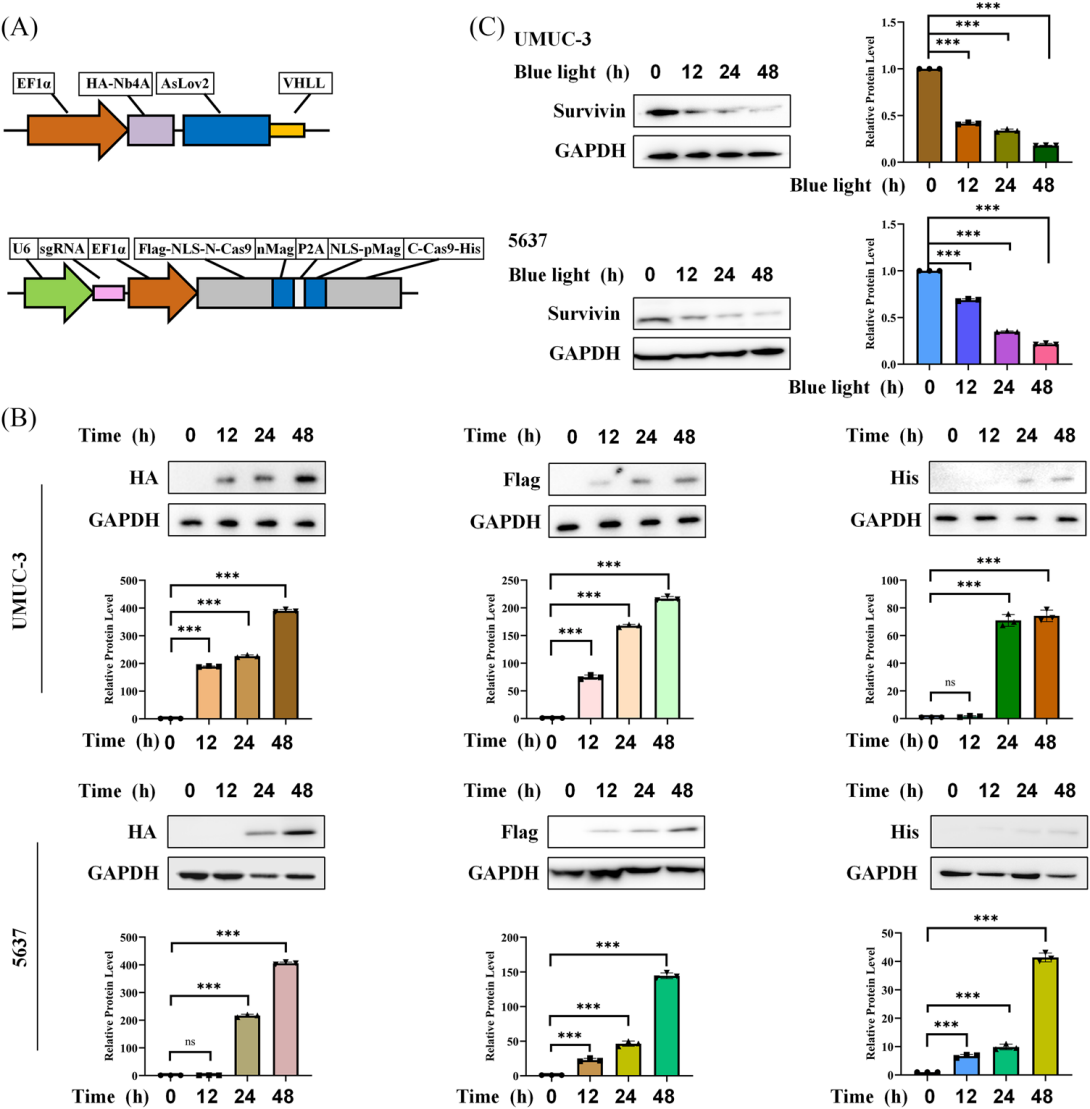
Combined application of paProtacL‐Survivin and paCas9‐Survivin degraded Survivin protein. (A) Schematic diagram of vector construction of paProtacL‐Survivin and paCas9‐Survivin. (B) Protein expression levels of each component were measured by western blot within 48 h after co‐transfection of UMUC‐3 and 5637 cells with paProtacL‐Survivin and paCas9‐Survivin. (C) paProtacL‐Survivin and paCas9‐Survivin were co‐transfected with UMUC‐3 and 5637 cells for 48 h, followed by blue‐light irradiation for 48 h, and then the expression level of Survivin protein was detected by western blot. MOI = 20.

Using the paProtacL‐Survivin and paCas9‐Survivin systems alone could effectively reduce the expression level of Survivin protein, but it was not clear what function they could play together. Tag‐antibodies were used to detect the expression of various components (Figure 4A) such as HA‐Nb4A‐AsLov2‐VHLL. The expression level of related proteins was detected within 48 h after co‐transfection of lentivirus, and western blot was detected by HA, Flag and His antibodies. The results indicated that related proteins levels gradually increased within 48 h (Figure 4B). At the same time, the cells were exposed to blue light after 48 h of co‐transfection with lentivirus. This phenomenon enabled genes to be expressed in large quantities and accumulate within cells. Then, it functions under the action of blue light. Samples were taken within 48 h of light to evaluate the Survivin protein expression level in the treated UMUC‐3 and 5637 cells. The results revealed that the expression level of Survivin protein progressively reduced with the addition of blue‐light exposure time, reaching the lowest level at 48 h (Figure 4C).

### 3.5 Degradation of the Survivin protein promoted apoptosis of bladder cancer cells in vitro

**FIGURE 5:**
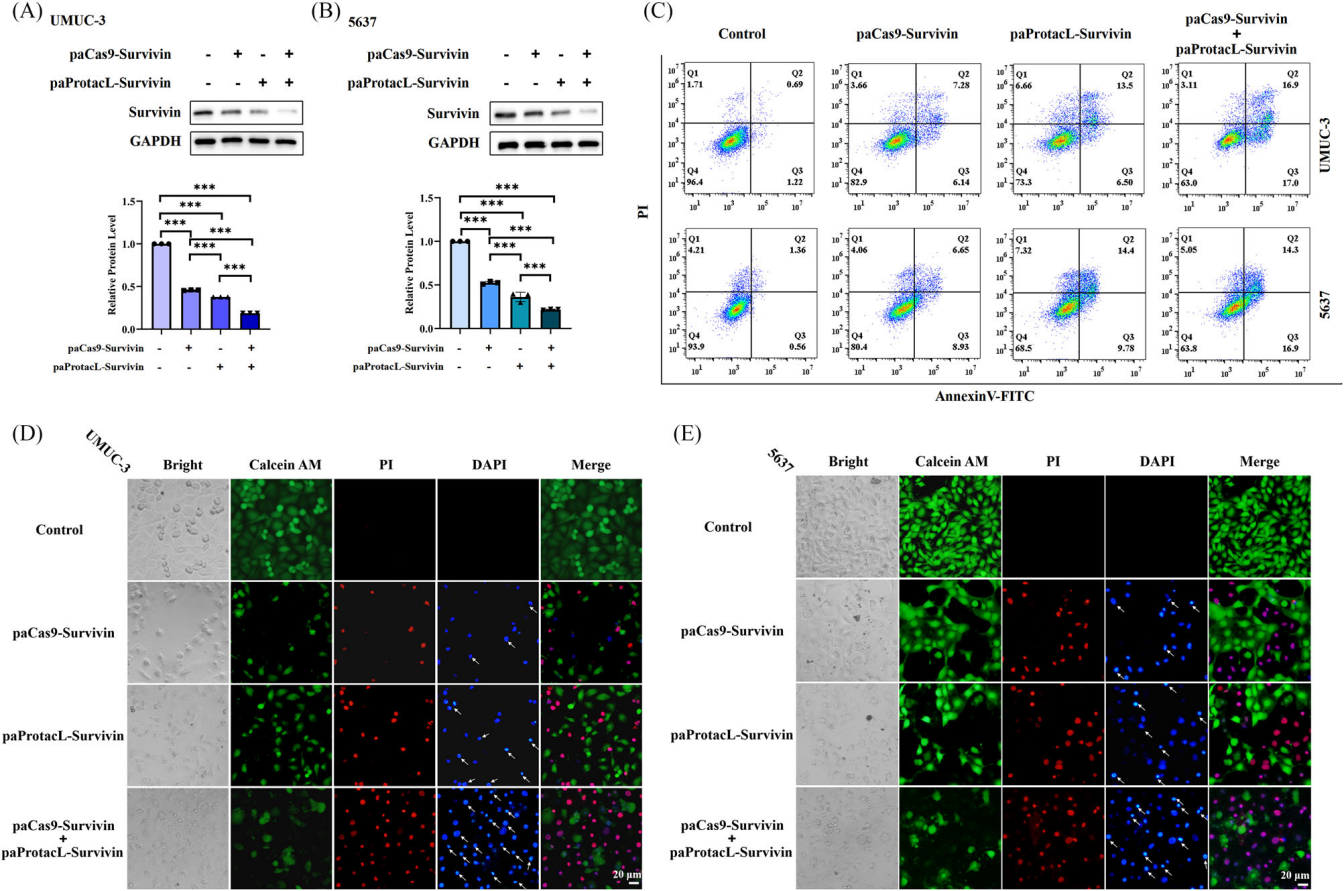
Inhibition of Survivin expression promoted apoptosis of UMUC‐3 and 5637 cells in vitro. (A and B) paProtacL‐Survivin and paCas9‐Survivin were transfected with UMUC‐3 and 5637 cells alone or in combination for 48 h, followed by 48 h of blue‐light irradiation, and the cells were collected, and the expression levels of Survivin protein were measured by western blot (MOI = 10). (C) Analysis of apoptosis levels of UMUC‐3 and 5637 cells after different treatments by flow cytometry (MOI = 20). (D and E) Fluorescence of UMUC‐3 and 5637 cells stained with calcein/PI/DAPI after different treatments were observed by fluorescence microscopy (MOI = 20). The arrow pointed to nuclear fragmentation and condensation. Scale bar: 20 µm. ‘+’ meant yes, ‘‐’ meant no.

Survivin, as an anti‐apoptotic protein, depicts an essential effect in the apoptosis of cancerous cells. Degradation of the Survivin protein could propagate the apoptosis of cancerous cells. For a more visual comparison of the differences between a single system and a dual system, the paProtacL‐Survivin and paCas9‐Survivin lentivirus systems were treated separately or jointly with UMUC‐3 (Figure 5A) and 5637 (Figure 5B) cells according to MOI = 10 for 48 h. Then the cells were irradiated with blue light for 48 h. The collected cells were tested for western blot. The results demonstrated that combining the two was more efficient in degrading Survivin than when used alone. The following experiment restored MOI = 20. Flow cytometry analysis showed that combining the two could boost the apoptosis of bladder cancer cells. The apoptosis rate of UMUC‐3 cells reached 33.9%, and that of 5637 cells reached 31.2% (Figure 5C) under the combined. Furthermore, the treated UMUC‐3 (Figure 5D) and 5637 (Figure 5E) cells were detected with calcein/PI/DAPI and the fluorescence of cells under different excitations was observed by fluorescence microscope. According to the instructions, green fluorescence symbolizes living cells, red fluorescence signifies dead cells and blue fluorescence depicts the DNA state in the nucleus (low concentration of DAPI is not easy to enter living cells in a short time, while it is easy to enter cells with damaged cell membranes). Through different fluorescence analyses, combined use could promote cell apoptosis more than single use, reduce living cells, increase dead cells and change the nuclear morphology of more cells, resulting in nuclear fragmentation and condensation.

### 3.6 Reduction of the Survivin protein inhibited the proliferation and migration of bladder cancer cells in vitro

**FIGURE 6:**
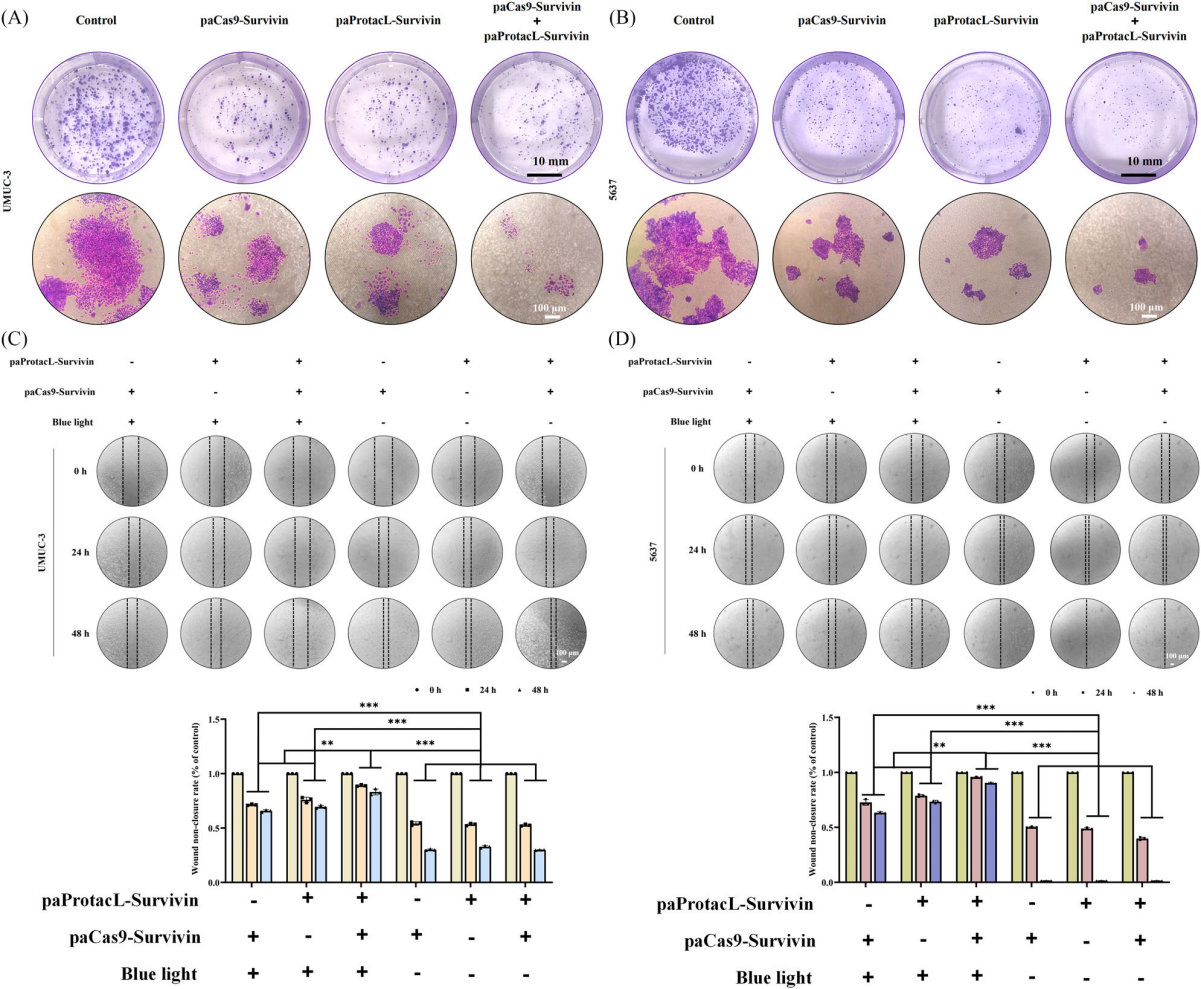
Degradation of Survivin protein inhibited the migration and proliferation of UMUC‐3 and 5637 cells in vitro. (A and B) UMUC‐3 and 5637 cells were treated with paProtacL‐Survivin and paCas9‐Survivin alone or in combination for 48 h, followed by 48 h of blue‐light irradiation. After 2 weeks, the cells were stained with crystal violet, and the colonies were photographed using a digital camera. The scale bar is 10 mm and 100 µm, respectively. (C and D) Representative images of UMUC‐3 and 5637 cell migration were assessed by wound healing assay. UMUC‐3 and 5637 cells were treated with paProtacL‐Survivin and paCas9‐Survivin alone or in combination for 48 h before scratching and then irradiated with blue light and photographed at 0, 24 and 48 h. MOI = 20.

Survivin not only exhibits an anti‐apoptotic role in cancer cells, but also inhibits the proliferation and migration of cancer cells.^34^ In the cloning formation assay, UMUC‐3 (Figure 6A) and 5637 (Figure 6B) cells were transfected with different lentivirus particles for 48 h, then exposed to blue light for another 48 h, then replaced with fresh medium and then changed the medium every 2 days, until 14 days later, crystal violet staining was performed. The results indicated that combined use showed more potent inhibition than single use. Similarly, in the cell scratch test, combined use in UMUC‐3 (Figure 6C) and 5637 (Figure 6D) cells still showed a strong ability to inhibit migration compared with the single use. The results above indicated that inhibition of Survivin expression reduced the proliferation and migration of bladder carcinoma cells, as well as the combination of paProtacL‐Survivin and paCas9‐Survivin systems could maximize the suppression of proliferation and migration.

### 3.7 Reducing Survivin protein inhibited tumour growth in vivo

**FIGURE 7:**
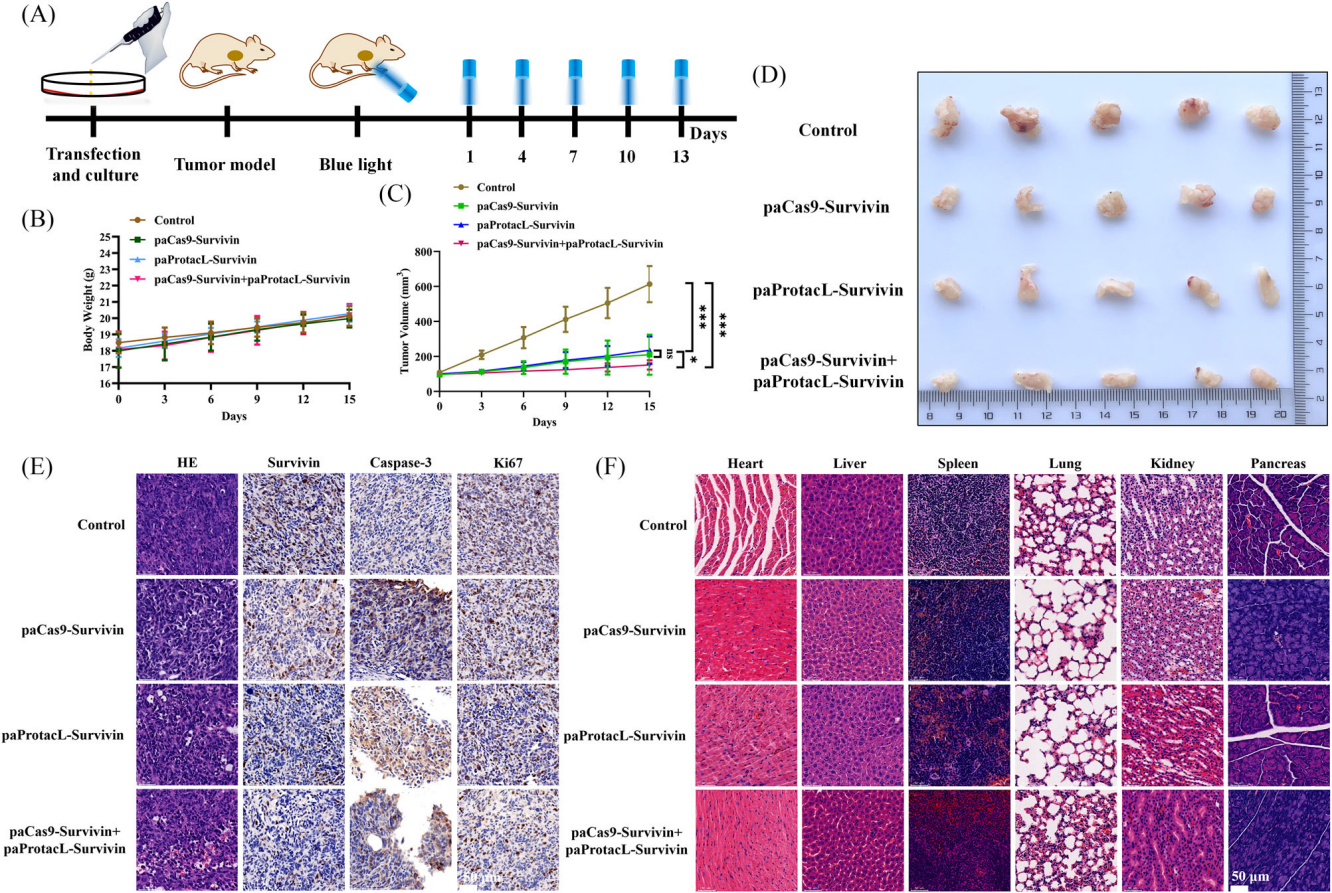
Reduced expression of Survivin protein inhibited tumour growth in vivo. (A) Schematic diagram of animal experimentation. (B) Weighing mice in the control and experimental groups during blue light irradiation. (C) Tumour volumes were measured in control and experimental mice during blue‐light irradiation. (D) Tumours from control and experimental mice were peeled and photographed 15 days after treatment. (E) HE and IHC analysis of tumours in the control and experimental groups. (F) HE staining of the main organs of mice in the control and experimental groups including heart, liver, spleen, lung, kidney and pancreas.

To verify the efficacy of paProtacL‐Survivin and/or paCas9‐Survivin systems, the CDX model was constructed for animal experiments. The experimental procedure is shown in Figure 7A. The mice were weighed and the tumour volumes were measured before each blue light irradiation. Body weight measurements revealed a slow increase in the body weight of these mice, indicating that the system was less toxic and did not affect the growth of the mice (Figure 7B). Measurement of tumour volumes showed that either the paProtacL‐Survivin or paCas9‐Survivin system could inhibit tumour growth after blue‐light irradiation and that the combination of the two was more effective (Figure 7C). Such results could also be seen in Figure 7D. Meanwhile, the results of HE and IHC on the tumour blocks of the experimental and control groups demonstrated that paProtacL‐Survivin and/or paCas9‐Survivin systems effectively reduced Survivin protein expression. Similarly, that inhibition of Survivin led to the accumulation of Caspase‐3, which in turn promoted apoptosis. Downregulation of Ki67 protein implied a decrease in tumour proliferation capacity (Figure 7E). Also, the HE staining showed that the heart, liver, spleen, lung, kidney and pancreas in the experimental group were comparable to those in the control group, with no significant histomorphology changes (Figure 7F). Overall, animal studies indicated that the combination of paProtacL‐Survivin and paCas9‐Survivin had the most robust ability to reduce Survivin protein and to inhibit tumour growth than either paProtacL‐Survivin or paCas9‐Survivin alone.

## 4 DISCUSSION

Multiple targets have been found too hard to be a drug in the cancer treatment process, and traditional small molecule drugs have difficulty in targeting ‘non‐druggable’ targets, specifically because these protein targets lack ‘pockets’ where they can bind to small molecule drugs.^13^, ^16^, ^18^, ^19^ Based on the simulation of PROTACs, we chose the nanobodies as the ‘warhead’ for binding the target protein.^7^, ^38^ VHL also was selected for the ligands of E3 ligases,^44^ and its high expression in bladder cancer compared to normal tissues was found by comparing TCGA and the human protein atlas database (Supporting Information Figure S2). And several PROTACS drugs using VHL E3 ligases are already in the clinical stage. The seven‐amino acid sequence ALAPYIP was the minimum recognition domain for the VHL and was selected as the ligand for E3.^26^, ^27^ Given the properties of the blue‐light receptor AsLov2,^24^, ^45^ a mock PROTACs model consisting of these three components forms the system of light‐induced protein degradation.^46^, ^47^ Such protein degradation systems that rely on the action of nanobodies have been partially reported. For example, Ibrahim et al.^7^ expressed nanobodies fused to the RING structural domain of E3 ligase RNF4 to construct a nanobody‐targeted degradation system of endogenous proteins. Also, the use of the LOV2 domain for photo‐controlled degradation has been studied. Hermann et al.^45^ fused cODC1 (C‐terminus of mouse ornithine decarboxylase) to the C‐terminus of AtLov2 (from *Arabidopsis thaliana*) and connected exogenous protein to the N‐terminus, the degradation of exogenous protein could be controlled by blue‐light irradiation. Combining the advantages of these studies, the pProtacL system was developed to provide rapid, specific and controlled degradation of endogenous proteins.

The paCas9 system referenced Nihongaki's technology, except that the dual plasmid system was changed to a single plasmid, and a lentiviral delivery system was used instead.^21^ Compared with the Nihongaki system, the advantage of this study was that transfection by lentivirus could increase the transfection efficiency of target genes. The system also took advantage of the ability of the blue‐light receptor protein nMag/pMag to form heterodimers for controlled editing of target genes. This work added a controlled approach compared to that reported by Qi et al.,^34^ which reduced the genotoxicity of intact Cas9 to some extent. Optimizing the selection of sgRNAs and mining for less toxic Cas proteins could help address the off‐target and toxic effects associated with the CRISPR‐Cas9 system,^43^, ^48^ or using Cas9 mRNA to prevent random insertions into the genome.^48^ Rosenblum et al.^49^ used lipid nanoparticles to effectively deliver Cas9 mRNA and sgRNAs to edit the *PLK1* gene. Meanwhile, Yu et al.^50^ developed a far‐red light‐activated split‐Cas9 system and successfully edited the *PLK1* gene in a mouse xenograft tumour model. Both paProtacL and paCas9 alone exert inhibitory functions on target genes, while the combination can compensate for each other's defects.

**FIGURE 8:**
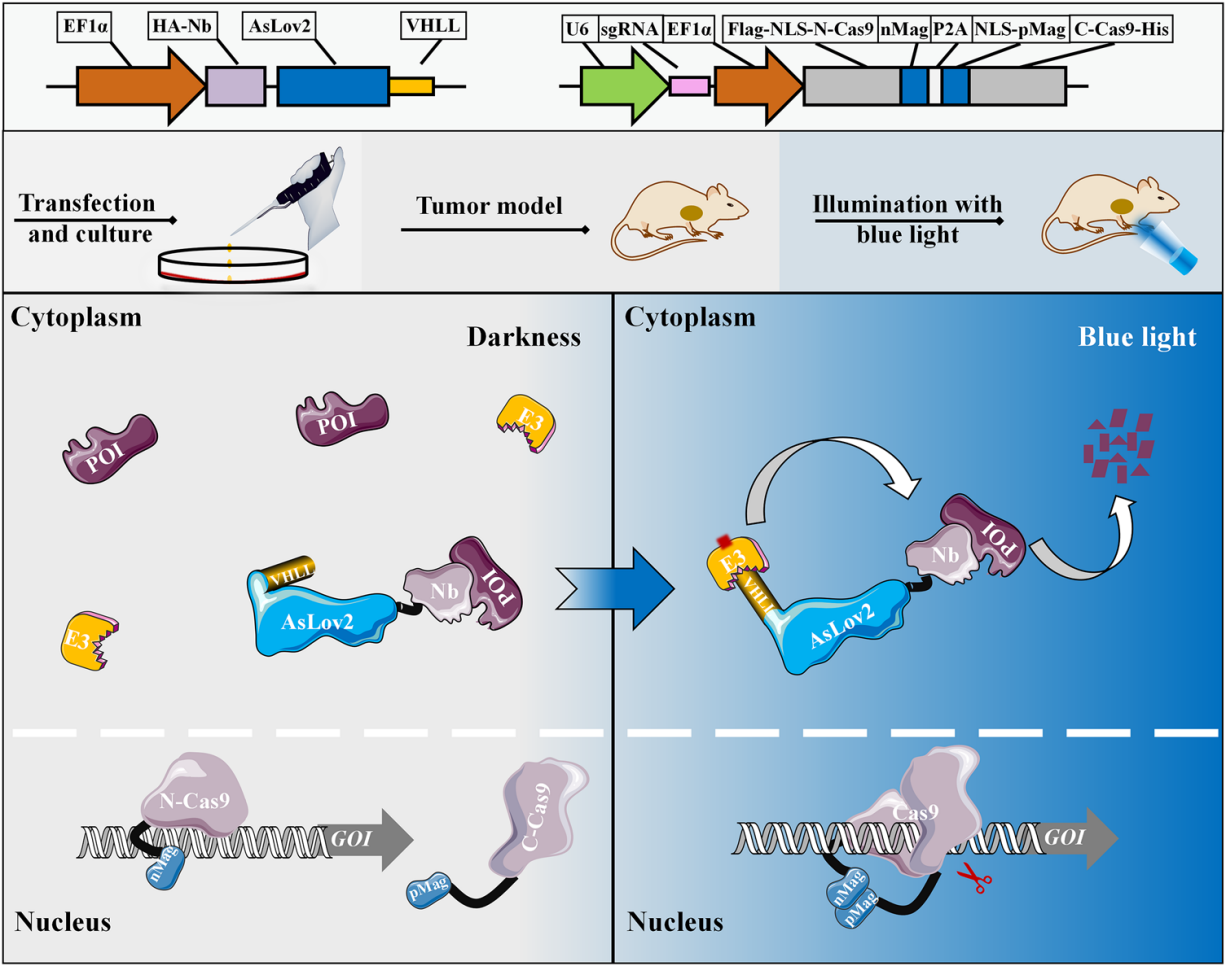
Pattern diagram of paProtacL combined with paCas9 to inhibit target protein expression. Above were paProtacL‐POI and paCas9‐GOI lentiviral vector constructs, respectively. In the middle were the animal procedures, including lentiviral transfection and cell culture, tumour implantation in nude mice and blue‐light treatment. In the large square below, the grey area represented the state of each component under darkness, with Nb‐specific binding of POI in the cytoplasm and the C‐terminus of AsLov2 hiding VHLL. The separated Cas9 proteins were free in the nucleus. The light blue area represented the state of the individual components under blue‐light irradiation. The C‐terminus of AsLov2 in the cytoplasm became loose, exposing VHLL, which attracted E3 ligase and allowed POI to be degraded by ubiquitination. In the nucleus, the separated Cas9 proteins also merge into one with the combination of nMag and pMag, thus, allowing controlled editing of GOI. Ultimately, the combined application of the two systems could minimize the expression of the target gene. GOI, Gene of interest; Nb, Nanobody; POI, Protein of interest; VHLL, the ligand of von Hippel‐Lindau.

Photo‐controlled gene editing and light‐induced protein degradation systems have great potential for treating human diseases.^51^ The process involves introducing the components of the photocontrol system or cells containing the therapeutic gene into the human body and using light to induce the degradation of the target gene at the transcriptional and protein levels, thereby achieving a therapeutic effect.^52^, ^53^ In this study, we combined the paProtacL and paCas9 systems (Figure 8). We found that they were more effective in degrading Survivin levels than alone and that the combination promoted apoptosis and inhibited migration and proliferation of bladder cancer cells in vitro. Also, animal study results demonstrated the effectiveness and utility of the system and that reducing Survivin expression inhibited tumour growth. Indeed, consideration of ways to enhance the penetration of light to penetrate thicker tissues is necessary for future study. The development of this system provides an effective strategy for studying proteins with unknown functions, while reducing the level of abnormally expressed genes in cancer can also serve the purpose of cancer therapy.

## CONFLICT OF INTEREST STATEMENT

The authors declare no conflict of interests.

## FUNDING INFORMATION

This study was supported by the National Key Research and Development Project of China (2018YFA0902804). And the National Natural Science Foundation (31670944, 81673345 and 31870861), the Science and Technology Innovation Action Plan of Shanghai (17431904600).

## Supporting information

Supporting Information

## Data Availability

The data that support the findings of this study are available from the corresponding author upon reasonable request.

## References

[1] Wang L , Karpova A , Gritsenko MA , et al. Proteogenomic and metabolomic characterization of human glioblastoma. Cancer Cell. 2021;39(4):509‐528. doi:10.1016/j.ccell.2021.01.006 33577785 PMC8044053

[2] Cao L , Huang C , Zhou DC , et al. Proteogenomic characterization of pancreatic ductal adenocarcinoma. Cell. 2021;184(19):5031‐5052. doi:10.1016/j.cell.2021.08.023 34534465 PMC8654574

[3] Satpathy S , Krug K , Beltran PMJ , et al. A proteogenomic portrait of lung squamous cell carcinoma. Cell. 2021;184(16):4348‐4371. doi:10.1016/j.cell.2021.07.016 34358469 PMC8475722

[4] Muñoz‐Hernández H , Pal M , Rodríguez CF , et al. Structural mechanism for regulation of the AAA‐ATPases RUVBL1‐RUVBL2 in the R2TP co‐chaperone revealed by cryo‐EM. Sci Adv. 2019;5(5):eaaw1616. doi:10.1126/sciadv.aaw1616 31049401 PMC6494491

[5] Clift D , McEwan WA , Labzin LI , et al. A method for the acute and rapid degradation of endogenous proteins. Cell. 2017;171(7):1692‐1706. doi:10.1016/j.cell.2017.10.033 29153837 PMC5733393

[6] Doudna JA , Charpentier E . Genome editing. The new frontier of genome engineering with CRISPR‐Cas9. Science. 2014;346(6213):1258096. doi:10.1126/science.1258096 25430774

[7] Ibrahim AFM , Shen L , Tatham MH , et al. Antibody RING‐mediated destruction of endogenous proteins. Mol Cell. 2020;79(1):155‐166. doi:10.1016/j.molcel.2020.04.032 32454028 PMC7332993

[8] Álvarez MM , Biayna J , Supek F . TP53‐dependent toxicity of CRISPR/Cas9 cuts is differential across genomic loci and can confound genetic screening. Nat Commun. 2022;13(1):4520. doi:10.1038/s41467-022-32285-1 35927263 PMC9352712

[9] Tao J , Wang Q , Mendez‐Dorantes C , Burns KH , Chiarle R . Frequency and mechanisms of LINE‐1 retrotransposon insertions at CRISPR/Cas9 sites. Nat Commun. 2022;13(1):3685. doi:10.1038/s41467-022-31322-3 35760782 PMC9237045

[10] Tao J , Bauer DE , Chiarle R . Assessing and advancing the safety of CRISPR‐Cas tools: From DNA to RNA editing. Nat Commun. 2023;14(1):212. doi:10.1038/s41467-023-35886-6 36639728 PMC9838544

[11] Kosicki M , Tomberg K , Bradley A . Repair of double‐strand breaks induced by CRISPR‐Cas9 leads to large deletions and complex rearrangements. Nat Biotechnol. 2018;36(8):765‐771. doi:10.1038/nbt.4192 30010673 PMC6390938

[12] Hanlon KS , Kleinstiver BP , Garcia SP , et al. High levels of AAV vector integration into CRISPR‐induced DNA breaks. Nat Commun. 2019;10(1):4439. doi:10.1038/s41467-019-12449-2 31570731 PMC6769011

[13] Li X , Pu W , Zheng Q , Ai M , Chen S , Peng Y . Proteolysis‐targeting chimeras (PROTACs) in cancer therapy. Mol Cancer. 2022;21(1):99. doi:10.1186/s12943-021-01434-3 35410300 PMC8996410

[14] Marei H , Tsai W‐TK , Kee Y‐S , et al. Antibody targeting of E3 ubiquitin ligases for receptor degradation. Nature. 2022;610(7930):182‐189. doi:10.1038/s41586-022-05235-6 36131013 PMC9534761

[15] Paiva S‐L , Crews CM . Targeted protein degradation: Elements of PROTAC design. Curr Opin Chem Biol. 2019;50:111‐119. doi:10.1016/j.cbpa.2019.02.022 31004963 PMC6930012

[16] Pettersson M , Crews CM . PROteolysis TArgeting Chimeras (PROTACs)—Past, present and future. Drug Discov Today Technol. 2019;31:15‐27. doi:10.1016/j.ddtec.2019.01.002 31200855 PMC6578591

[17] Swatek KN , Komander D . Ubiquitin modifications. Cell Res. 2016;26(4):399‐422. doi:10.1038/cr.2016.39 27012465 PMC4822133

[18] Li Ke , Crews CM . PROTACs: Past, present and future. Chem Soc Rev. 2022;51(12):5214‐5236. doi:10.1039/d2cs00193d 35671157 PMC10237031

[19] Qi S , Dong J , Xu Z‐Y , Cheng X , Zhang W , Qin J . PROTAC: An effective targeted protein degradation strategy for cancer therapy. Front Pharmacol. 2021;12:692574. doi:10.3389/fphar.2021.692574 34025443 PMC8138175

[20] Liu Y , Zeng Y , Liu Li , et al. Synthesizing AND gate genetic circuits based on CRISPR‐Cas9 for identification of bladder cancer cells. Nat Commun. 2014;5:5393. doi:10.1038/ncomms6393 25373919

[21] Nihongaki Y , Kawano F , Nakajima T , Sato M . Photoactivatable CRISPR‐Cas9 for optogenetic genome editing. Nat Biotechnol. 2015;33(7):755‐760. doi:10.1038/nbt.3245 26076431

[22] Häusser M . Optogenetics: The age of light. Nat Methods. 2014;11(10):1012‐1014. doi:10.1038/nmeth.3111 25264778

[23] Shao J , Wang M , Yu G , et al. Synthetic far‐red light‐mediated CRISPR‐dCas9 device for inducing functional neuronal differentiation. Proc Nat Acad Sci USA. 2018;115(29):E6722‐E6730. doi:10.1073/pnas.1802448115 29967137 PMC6055150

[24] Lungu OI , Hallett RA , Choi EJ , Aiken MJ , Hahn KM , Kuhlman B . Designing photoswitchable peptides using the AsLOV2 domain. Chem Biol. 2012;19(4):507‐517. doi:10.1016/j.chembiol.2012.02.006 22520757 PMC3334866

[25] Strickland D , Lin Y , Wagner E , et al. TULIPs: Tunable, light‐controlled interacting protein tags for cell biology. Nat Methods. 2012;9(4):379‐384. doi:10.1038/nmeth.1904 22388287 PMC3444151

[26] Jr JSS , Fonseca FN , Koldobskiy M , et al. Chemical genetic control of protein levels: Selective in vivo targeted degradation. J Am Chem Soc. 2004;126(12):3748‐3754. doi:10.1021/ja039025z 15038727

[27] Chu T , Gao Na , Li Q , et al. Specific knockdown of endogenous Tau protein by peptide‐directed ubiquitin‐proteasome degradation. Cell Chem Biol. 2016;23(4):453‐461. doi:10.1016/j.chembiol.2016.02.016 27105281

[28] Niopek D , Benzinger D , Roensch J , et al. Engineering light‐inducible nuclear localization signals for precise spatiotemporal control of protein dynamics in living cells. Nat Commun. 2014;5:4404. doi:10.1038/ncomms5404 25019686 PMC4104460

[29] Prozzillo Y , Fattorini G , Santopietro MV , et al. Targeted protein degradation tools: Overview and future perspectives. Biology (Basel). 2020;9(12):421. doi:10.3390/biology9120421 33256092 PMC7761331

[30] Liu M , Li L , Jin D , Liu Y . Nanobody: A versatile tool for cancer diagnosis and therapeutics. Wiley Interdiscip Rev Nanomed Nanobiotechnol. 2021;13(4):e1697. doi:10.1002/wnan.1697 33470555

[31] Salvesen GS , Duckett CS . IAP proteins: Blocking the road to death's door. Nat Rev Mol Cell Biol. 2002;3(6):401‐410. doi:10.1038/nrm830 12042762

[32] Deng C , Hu F , Zhao Z , et al. The establishment of quantitatively regulating expression cassette with sgRNA targeting BIRC5 to elucidate the synergistic pathway of Survivin with P‐Glycoprotein in cancer multi‐drug resistance. Front Cell Dev Biol. 2021;9:797005. doi:10.3389/fcell.2021.797005 35047507 PMC8762277

[33] Hu F , Deng C , Zhou Y , et al. Multistage targeting and dual inhibiting strategies based on bioengineered tumor matrix microenvironment‐mediated protein nanocages for enhancing cancer biotherapy. Bioeng Transl Med. 2022;7(2):e10290. doi:10.1002/btm2.10290 35600646 PMC9115700

[34] Qi Y , Liu Y , Yu B , et al. A lactose‐derived CRISPR/Cas9 delivery system for efficient genome editing in vivo to treat orthotopic hepatocellular carcinoma. Adv Sci. 2020;7(17):2001424. doi:10.1002/advs.202001424 PMC7507475 32995132

[35] Wang X , He Z . Lentiviral vectors for gene therapy. Encycloped Life Sci. 2022:1‐10. doi:10.1002/9780470015902.a0029449

[36] Wang X , Ma C , Labrada RR , Zhou Qin TX , He Z , Wei Y . Recent advances in lentiviral vectors for gene therapy. Sci China Life Sci. 2021;64(11):1842‐1857. doi:10.1007/s11427-021-1952-5 34708326

[37] Nicolas CT , VanLith CJ , Hickey RD , et al. In vivo lentiviral vector gene therapy to cure hereditary tyrosinemia type 1 and prevent development of precancerous and cancerous lesions. Nat Commun. 2022;13(1):5012. doi:10.1038/s41467-022-32576-7 36008405 PMC9411607

[38] Zhang N , Guo H , Zheng W , Wang T , Ma X . Design and screening of a chimeric survivin‐specific nanobody and its anticancer activities in vitro. Anticancer Drugs. 2016;27(9):839‐847. doi:10.1097/CAD.0000000000000394 27362789

[39] Jensen EC . Quantitative analysis of histological staining and fluorescence using ImageJ. Anatom Record (Hoboken). 2013;296(3):378‐381. doi:10.1002/ar.22641 23382140

[40] Zhang Z , Wang Y , Ding Y , Hattori M . Structure‐based engineering of anti‐GFP nanobody tandems as ultra‐high‐affinity reagents for purification. Sci Rep. 2020;10(1):6239. doi:10.1038/s41598-020-62606-7 32277083 PMC7148334

[41] Li XY , Li T , Li XJ , Wang JN , Chen Z . TSG‐6 induces apoptosis of human hypertrophic scar fibroblasts via activation of the Fas/FasL signalling pathway. Folia Biologica (Praha). 2018;64(5‐6):173‐181. 10.14712/fb2018064050173 30938674

[42] Chen S , Deng C , Zheng W , et al. Cannabidiol effectively promoted cell death in bladder cancer and the improved intravesical adhesion drugs delivery strategy could be better used for treatment. Pharmaceutics. 2021;13(9):1415. doi:10.3390/genes1030413 34575494 PMC8471856

[43] Pausch P , Al‐Shayeb B , Bisom‐Rapp E , et al. CRISPR‐CasΦ from huge phages is a hypercompact genome editor. Science. 2020;369(6501):333‐337. 32675376 10.1126/science.abb1400 PMC8207990

[44] William GK Jr . Von Hippel‐Lindau disease: Insights into oxygen sensing, protein degradation, and cancer. J Clin Invest. 2022;132(18):e162480. doi:10.1172/JCI162480 36106637 PMC9479583

[45] Hermann A , Liewald JF , Gottschalk A . A photosensitive degron enables acute light‐induced protein degradation in the nervous system. Curr Biol. 2015;25(17):R749‐R750. doi:10.1016/j.cub.2015.07.040 26325132

[46] Ryan A , Liu J , Deiters A . Targeted protein degradation through fast optogenetic activation and its application to the control of cell signaling. J Am Chem Soc. 2021;143(24):9222‐9229. doi:10.1021/jacs.1c04324 34121391

[47] Sun W , Zhang W , Zhang C , et al. Light‐induced protein degradation in human‐derived cells. Biochem Biophys Res Commun. 2017;487(2):241‐246. doi:10.1016/j.bbrc.2017.04.041 28412349

[48] Naddaf M . The science events to watch for in 2023. Nature. 2023;613(7942):11‐12. doi:10.1038/d41586-022-04444-3 36536125

[49] Rosenblum D , Gutkin A , Kedmi R , et al. CRISPR‐Cas9 genome editing using targeted lipid nanoparticles for cancer therapy. Sci Adv. 2020;6(47):eabc9450. doi:10.1126/sciadv.abc9450 33208369 PMC7673804

[50] Yu Y , Wu X , Guan N , et al. Engineering a far‐red light‐activated split‐Cas9 system for remote‐controlled genome editing of internal organs and tumors. Sci Adv. 2020;6(28):eabb1777. doi:10.1126/sciadv.abb1777 32923591 PMC7455487

[51] Guan N , Gao X , Ye H . Engineering of optogenetic devices for biomedical applications in mammalian synthetic biology. Eng Biol. 2022;6(2‐3):35‐49. doi:10.1049/enb2.12022 36969102 PMC9996731

[52] Gao TT , Oh T‐J , Mehta K , et al. The clinical potential of optogenetic interrogation of pathogenesis. Clin Transl Med. 2023;13(5):e1243. doi:10.1002/ctm2.1243 37132114 PMC10154842

[53] Malogolovkin A , Egorov AD , Karabelsky A , Ivanov RA , Verkhusha VV . Optogenetic technologies in translational cancer research. Biotechnol Adv. 2022;60:108005. doi:10.1016/j.biotechadv.2022.108005 35690273

