# Supplementary material for: Split‐Cas9‐based targeted gene editing and nanobody‐mediated proteolysis‐targeting chimeras optogenetically coordinated regulation of Survivin to control the fate of cancer cells
Source: Clin Transl Med. 2023 Aug 24;13(8):e1382. doi: 10.1002/ctm2.1382 (PMC10449816; doi:10.1002/ctm2.1382)
Supplement: Supplementary file 1 — Supporting Information [file CTM2-13-e1382-s001.docx]

Split-Cas9-based targeted gene editing and nanobody-mediated proteolysis-targeting chimeras optogenetically coordinated regulation of Survivin to control the fate of cancer cells

Running title: multi-level regulation of key factors to govern cancer cells fate

Changping Deng^1^, Shihui Li^1^, Yuping Liu^2^, Wen Bao^2^, Chengnan Xu^2^, Wenyun Zheng^2†^, Meiyan Wang^3^, Xingyuan Ma^1†^

1State Key Laboratory of Bioreactor Engineering, East China University of Science and Technology, Shanghai 200237, P. R. China

2Shanghai Key Laboratory of New Drug Design, School of Pharmacy, East China University of Science and Technology, Shanghai 200237, P. R. China

3Synthetic Biology and Biomedical Engineering Laboratory, Biomedical Synthetic Biology, Research Center, Shanghai Key Laboratory of Regulatory Biology, Institute

of Biomedical, Sciences and School of Life Sciences, East China Normal University, Shanghai, China

† To whom correspondence should be addressed.

Prof. Dr. Xingyuan Ma, E-mail: maxy@ecust.edu.cn

Prof. Dr. Wenyun Zheng, Email: zwy@ecust.edu.cn

Full postal: Laboratory of Biopharmaceutical and Cell Engineering, School of Biological, Engineering State Key Laboratory of Bioreactor Engineering, East China University of Science and Technology, 130 Meilong Road, P.O. Box No. 365, Shanghai, 200237, P. R. China

**Supporting Information**

**Figure S1.** Construction of UMUC-3-EGFP stable transfer cell line.

**Figure S2.** Bioinformatics analysis of VHL expression levels in cancers.

**Table S1.** Abbreviations and corresponding full names.

**Table S2.** The sgRNA and qRT-PCR primers used in this study.

**Table S3.** Amino acid sequences of each component of the vector were constructed in this study.


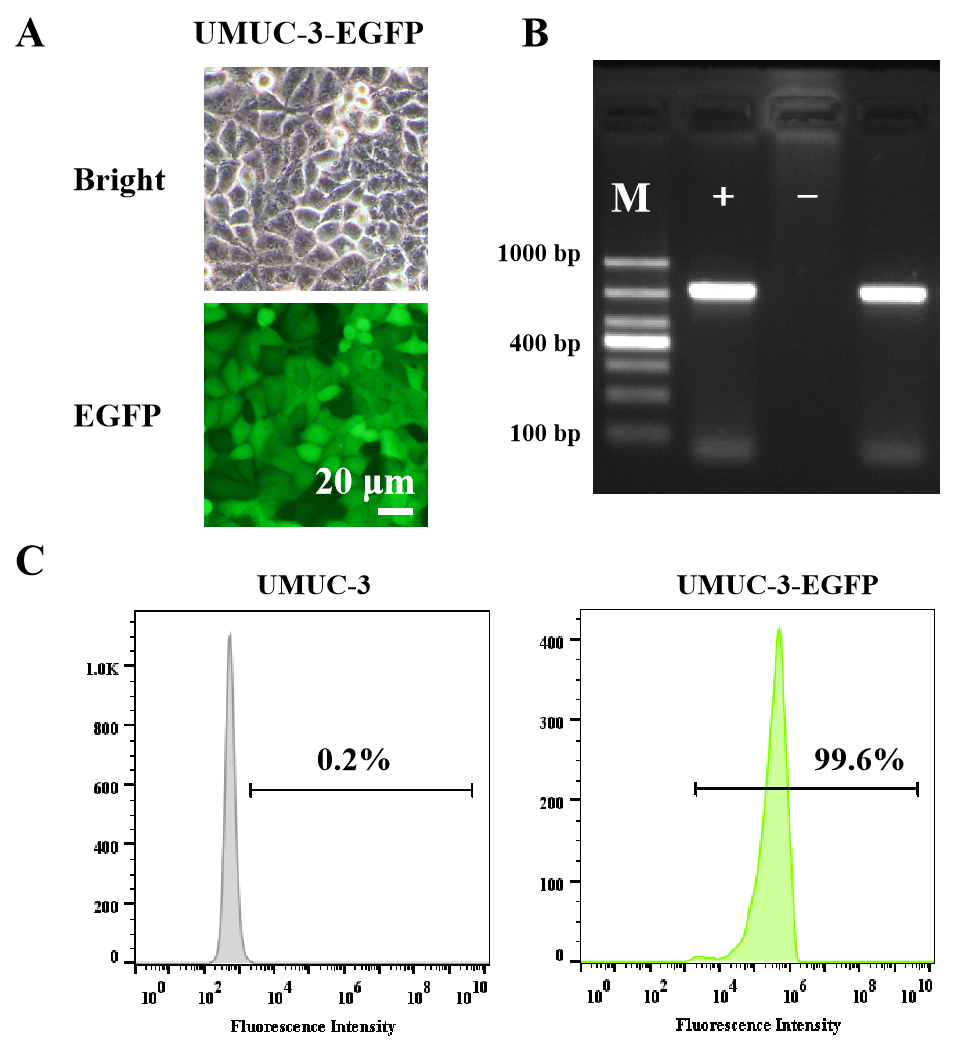


**Figure S1. Construction of UMUC-3-EGFP stable transfer cell line.** Construction of UMUC-3-EGFP stable transfer cell line. A: Fluorescence levels of UMUC-3-EGFP observed by fluorescence microscopy. B: PCR detection of EGFP expression on the UMUC-3-EGFP genome of the stable transfer cell line. C: Detection of fluorescent expression levels of UMUC-3 and UMUC-3-EGFP by flow cytometry. Scale bar: 20 μm.


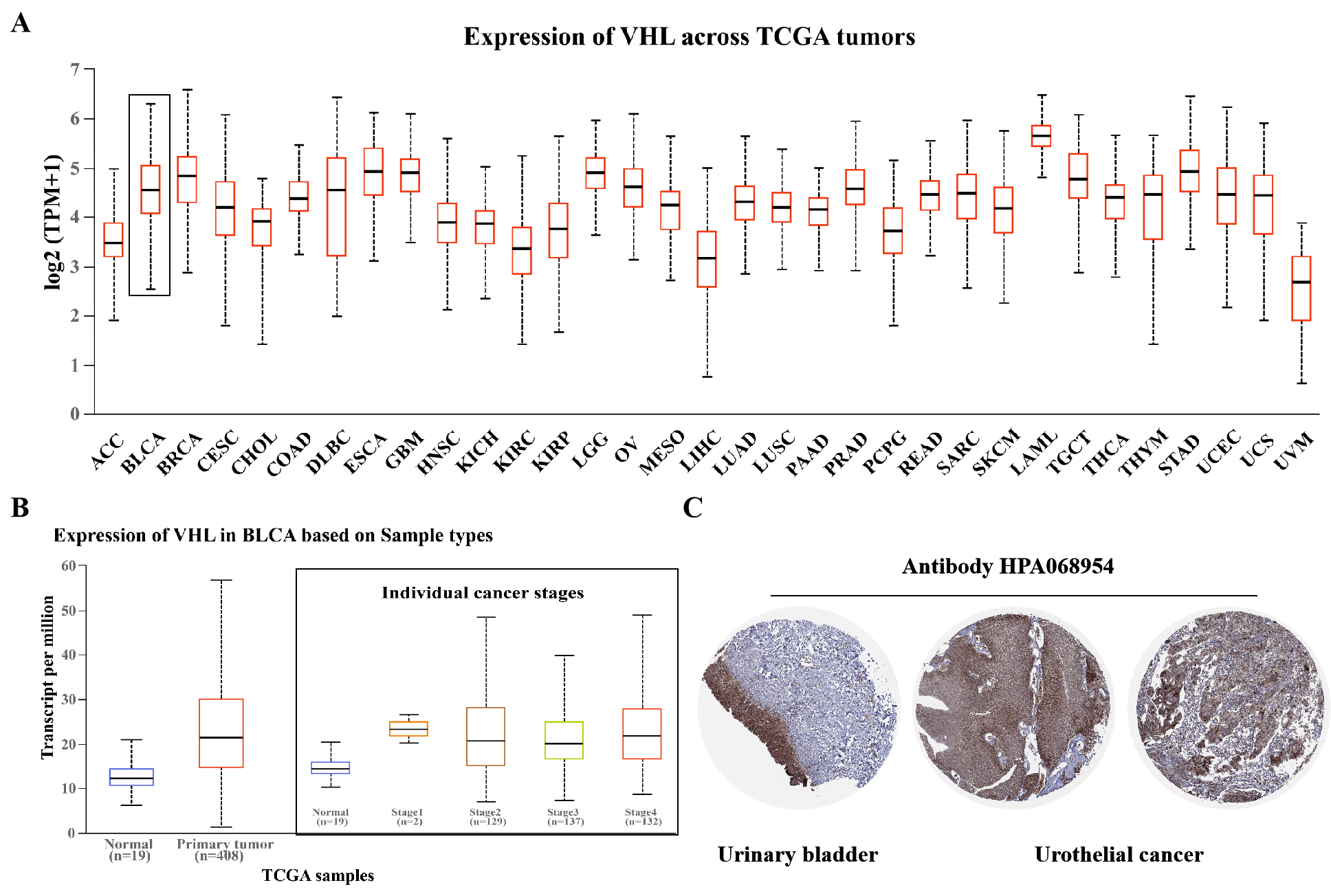


**Figure S2. Bioinformatics analysis of VHL expression levels in cancers.** Bioinformatics analysis of VHL expression levels in cancers. A: Analysis of VHL gene expression levels in pan-cancer by TCGA database. B: Analysis of VHL gene expression levels in bladder urothelial carcinoma based on sample types and individual cancer stages. C: Analysis of VHL protein expression levels under antibody staining by the human protein atlas database.

**Table S1.** Abbreviations and corresponding full names.

| Abbreviations | Full names |
| --- | --- |
| ACC | Adrenocortical carcinoma |
| BLCA | Bladder urothelial carcinoma |
| BRCA | Breast invasive carcinoma |
| CESC | Cervical squamous cell carcinoma |
| CHOL | Cholangiocarcinoma |
| COAD | Colon adenocarcinoma |
| DLBC | Diffuse large B-cell lymphoma |
| ESCA | Esophageal carcinoma |
| GBM | Glioblastoma multiforme |
| HNSC | Head and Neck squamous cell carcinoma |
| KICH | Kidney chromophobe |
| KIRC | Kidney renal clear cell carcinoma |
| KIRP | Kidney renal papillary cell carcinoma |
| LGG | Brain lower grade glioma |
| OV | Ovarian serous cystadenocarcinoma |
| MESO | Mesothelioma |
| LIHC | Liver hepatocellular carcinoma |
| LUAD | Lung adenocarcinoma |
| LUSC | Lung squamous cell carcinoma |
| PAAD | Pancreatic adenocarcinoma |
| PRAD | Prostate adenocarcinoma |
| PCPG | Pheochromocytoma and Paraganglioma |
| READ | Rectum adenocarcinoma |
| SARC | Sarcoma |
| SKCM | Skin cutaneous melanoma |
| AML | Acute Myeloid Leukemia |
| TGCT | Testicular germ cell tumors |
| THCA | Thyroid carcinoma |
| THYM | Thymoma |
| STAD | Stomach adenocarcinoma |
| UCEC | Uterine corpus endometrial carcinoma |
| UCS | Uterine carcinosarcoma |
| UVM | Uveal Melanoma |

**Table S2.** The sgRNA and qRT-PCR primers used in this study.

| sgRNA-EGFP | F | 5’-GGCGAGGGCGATGCCACCTA-3’ |
| --- | --- | --- |
|  | R | 5’-TAGGTGGCATCGCCCTCGCC-3’ |
| sgRNA-Survivin | F | 5’-CTGTCCCTTGCAGATGGCCG-3’ |
|  | R | 5’-CGGCCATCTGCAAGGGACAG-3’ |
| Survivin-qRT-PCR | F | 5’-AAGAACTGGCCCTTCTTGGA-3’ |
|  | R | 5’-CAACCGGACGAATGCTTTT-3’ |
| EGFP-qRT-PCR | F | 5’-ACCCTCGTGACCACCCTGAC-3’ |
|  | R | 5’-TGTAGTTGCCGTCGTCCTTGAAG-3’ |
| β-actin-qRT-PCR | F | 5’-ATTGGCAATGAGCGGTTC-3’ |
|  | R | 5’-GGATGCCACAGGACTCCAT-3’ |

**Table S3.** Amino acid sequences of each component of the vector were constructed in this study.

| **HA-Nb4A-AsLov2-VHLL** |
| --- |
| MYPYDVPDYAQVQLQESGGGLVQPGGSLRLSCAASGRTFSAVPIGWFRQAPGKEREFVAA ISQLPFHHYYADSVKGRFTISRDNAKNTVYLQMNSLKPEDTAVYYCAASGVYKVAYDWQH WGQGTQVTVSSGSGLATTLERIEKNFVITDPRLPDNPIIFASDSFLQLTEYSREEILGRN CRFLQGPETDRATVRKIRDAIDNQTEVTVQLINYTKSGKKFWNVFHLQPMRDYKGDVQYF IGVQLDGTERLHGAAEREAVMLIKKTAFQIAEAKELPALAPYIP |
| **HA- LaG16-AsLov2-VHLL** |
| MYPYDVPDYAAQVQLVESGGRLVQAGDSLRLSCAASGRTFSTSAMAWFRQAPGREREFVA AITWTVGNTILGDSVKGRFTISRDRAKNTVDLQMDNLEPEDTAVYYCSARSRGYVLSVLR SVDSYDYWGQGTQVTVSSPPVATGSGLATTLERIEKNFVITDPRLPDNPIIFASDSFLQL TEYSREEILGRNCRFLQGPETDRATVRKIRDAIDNQTEVTVQLINYTKSGKKFWNVFHLQ PMRDYKGDVQYFIGVQLDGTERLHGAAEREAVMLIKKTAFQIAEAKELPALAPYIP |
| **Flag-NLS-N-Cas9-nMag-P2A-NLS-pMag-C-Cas9-His** |
| MDYKDHDGDYKDHDIDYKDDDDKMAPKKKRKVGIHGVPAADKKYSIGLDIGTNSVGWAVI TDEYKVPSKKFKVLGNTDRHSIKKNLIGALLFDSGETAEATRLKRTARRRYTRRKNRICY LQEIFSNEMAKVDDSFFHRLEESFLVEEDKKHERHPIFGNIVDEVAYHEKYPTIYHLRKK LVDSTDKADLRLIYLALAHMIKFRGHFLIEGDLNPDNSDVDKLFIQLVQTYNQLFEENPI NASGVDAKAILSARLSKSRRLENLIAQLPGEKKNGLFGNLIALSLGLTPNFKSNFDLAED AKLQLSKDTYDDDLDNLLAQIGDQYADLFLAAKNLSDAILLSDILRVNTEITKAPLSASM IKRYDEHHQDLTLLKALVRQQLPEKYKEIFFDQSKNGYAGYIDGGASQEEFYKFIKPILE KMDGTEELLVKLNREDLLRKQRTFDNGSIPHQIHLGELHAILRRQEDFYPFLKDNREKIE KILTFRIPYYVGPLARGNSRFAWMTRKSEETITPWNFEEVVDKGASAQSFIERMTNFDKN LPNEKVLPKHSLLYEYFTVYNELTKVKYVTEGMRKPAFLSGEQKKAIVDLLFKTNRKVTV KQLKEDYFKKIECFDSVEISGVEDRFNASLGTYHDLLKIIKDKDFLDNEENEDILEDIVL TLTLFEDREMIEERLKTYAHLFDDKVMKQLKRRRYTGWGRLSRKLINGIRDKQSGKTILD FLKSDGFANRNFMQLIHDDSLTFKEDIQKAQVGGGGSGGGGSGGGGSHTLYAPGGYDIMG YLDQIGNRPNPQVELGPVDTSCALILCDLKQKDTPIVYASEAFLYMTGYSNAEVLGRNCR FLQSPDGMVKPKSTRKYVDSNTINTIRKAIDRNAEVQVEVVNFKKNGQRFVNFLTIIPVR DETGEYRYSMGFQCETEEFGSGATNFSLLKQAGDVEENPGPPKKKRKVMHTLYAPGGYDI MGYLRQIRNRPNPQVELGPVDTSCALILCDLKQKDTPIVYASEAFLYMTGYSNAEVLGRN CRFLQSPDGMVKPKSTRKYVDSNTINTIRKAIDRNAEVQVEVVNFKKNGQRFVNFLTIIP VRDETGEYRYSMGFQCETEGGGGSGGGGSGGGGSEFSGQGDSLHEHIANLAGSPAIKKGI LQTVKVVDELVKVMGRHKPENIVIEMARENQTTQKGQKNSRERMKRIEEGIKELGSQILK EHPVENTQLQNEKLYLYYLQNGRDMYVDQELDINRLSDYDVDHIVPQSFLKDDSIDNKVL TRSDKNRGKSDNVPSEEVVKKMKNYWRQLLNAKLITQRKFDNLTKAERGGLSELDKAGFI KRQLVETRQITKHVAQILDSRMNTKYDENDKLIREVKVITLKSKLVSDFRKDFQFYKVRE INNYHHAHDAYLNAVVGTALIKKYPKLESEFVYGDYKVYDVRKMIAKSEQEIGKATAKYF FYSNIMNFFKTEITLANGEIRKRPLIETNGETGEIVWDKGRDFATVRKVLSMPQVNIVKK TEVQTGGFSKESILPKRNSDKLIARKKDWDPKKYGGFDSPTVAYSVLVVAKVEKGKSKKL KSVKELLGITIMERSSFEKNPIDFLEAKGYKEVKKDLIIKLPKYSLFELENGRKRMLASA GELQKGNELALPSKYVNFLYLASHYEKLKGSPEDNEQKQLFVEQHKHYLDEIIEQISEFS KRVILADANLDKVLSAYNKHRDKPIREQAENIIHLFTLTNLGAPAAFKYFDTTIDRKRYT STKEVLDATLIHQSITGLYETRIDLSQLGGDGSGHHHHHH |
